# Supplementary material for: The influence of care types on the development of orphans——An empirical study from China
Source: PLoS One. 2024 Oct 17;19(10):e0309669. doi: 10.1371/journal.pone.0309669 (PMC11486387; doi:10.1371/journal.pone.0309669)
Supplement: Attachment — Submitted filename: reply-plosone.docx [file pone.0309669.s002.docx]

**To the editor and reviewers:**

We were pleased to receive constructive comments from two anonymous reviewers and the editor. We appreciate a lot for all your efforts in reviewing this paper. These comments help us improve this paper. We have carefully considered the suggestions from two reviewers and modified the paper. In the following section, we respond to the comments of each reviewer point by point.

**Response to Reviewer#1:**

*This is a thorough review and all the statistical analysis data has been clearly presented. It gives a good sense of all the parameters used to perform the heterogenous analysis on different aspects of welfare and has not just focused on social participation.*

We express our sincere gratitude to the reviewer for providing insightful comments on our paper. Your feedback has been invaluable in enhancing the overall quality of this study. We have thoroughly adjusted various parts of the article and all modified parts are marked in red.

**Response to reviewer#2:**

***Abstract***
*1. Improve the abstract structure that contains the essence of the problem, research objectives, methodology, population & sample, data collection techniques using measuring instruments, data analysis techniques, results, conclusions, and recommendations.*

*2. Grammar and Style:*

*a. Change "placement types is a key factor" to "placement types are a key factor".*

*b. Change "grandparent care have a significant positive impact" to "grandparent care has a significant positive impact".*

*c. The sentence "and this impact is moderated by the social participation of guardians" could be clearer if rephrased to "**with this impact being moderated by the social participation of guardians."*

Thank you for providing valuable suggestions for refining the abstract of the article. These changes will enhance the article's alignment with current literature and improve its overall standardization.

Firstly, we've adopted your suggestion and rewrite the whole abstract according to the sequence of the essence of the problem, research objectives, methodology, population & sample, data collection techniques using measuring instruments, data analysis techniques, results, conclusions, and recommendations. All modified parts are marked in red(lines 9-21)

Secondly, we appreciate your attention to grammar and style problem. We have changed all the three expression errors and marked in red (lines 9,15,16).

*Detailing the Methodology:*

*a. Mention the sample size or provide a brief description of the survey used to give clearer context about the data.*

We thank the reviewer for this constructive suggestion. We have added a brief description of survey and marked it in red（lines 11-13）. The data sample consists of 320 valid samples of orphans and their families, including 166 boys and 154 girls. And，the data we used was based on cross-sectional survey related to orphans from LZ City, Sichuan Province, in 2020.

*b. Specify the data analysis techniques used, such as regression analysis, to add depth to the information.*

Thank you for your thorough review of the abstract and your valuable comments. We have rephrased the sentence and marked it in red（lines 13）to explain the analysis techniques we used. The study conducted multiple linear regression model to analyze the relationship between care types and other family variables.

*Linking Sentences:*

*Improve the connection between sentences. For example, "Based on the moderating effect of guardians' social participation" could be clarified how it leads to "this paper uses the survey data...".*

Thanks to the reviewer's suggestion, we have revised the text to express" *the moderating effect of guardians' social participation*" . The necessary changes have been implemented throughout the text. The modified parts are marked in red（lines 15）.

*Specifying Heterogeneity Tests:*

*Expand a bit on the "Heterogeneity tests" to explain why these tests were conducted and what was specifically measured.*

The core function of *the "Heterogeneity tests"* is to explore the differences between the variety of the groups. The results of inter group differences revealed that the effects of care types on orphan development are influenced by the gender and age of orphans. The modified parts are marked in red（lines 18-19）.

***Introduction***

*1. In lines 30-32, that is According to China's relevant policies and regulations, the placement types for these orphans mainly include kinship care, institutional care, family foster care and legal adoption. Can you further explain what is meant by kinship care, institutional care, family care and legal adoption.*

We sincerely appreciate the reviewer's thorough examination of our article. According to laws and cultural customs, the care types for these orphans mainly include kinship care, institutional care, family foster care and legal adoption.

Kinship care occurs when close relatives of the orphan, such as grandparents, siblings, or other extended family members, voluntarily take on the responsibility of raising the orphan when the parents are deceased or unable to provide care.

Institutional care refers to placing the orphan in a child welfare institution (such as an orphanage) established by the government or social organizations, where professionals provide daily care and education. This method is suitable when there are no relatives available to care for the orphan or when the relatives are unable to provide care.

Foster care involves placing the orphan in a foster family that has been carefully screened and trained, where foster parents provide care on behalf of the state. This method aims to provide the orphan with a family-like environment, promoting their psychological well-being and social adaptability.

Legal adoption refers to eligible families formally adopting the orphan through legal procedures, becoming their legal parents. After adoption, the adoptive family assumes the legal responsibility for the orphan's upbringing and education, and the orphan enjoys the same rights as biological children of the adoptive parents.

*2. State the main objectives of the research. What does the researcher aim to achieve with this study?*

We thank the reviewer for pointing out this. The question you raised is crucial to the article.

In 2022, China had more than 190,000 orphans. These children were placed in four different types of care. Each care types have different effects on the development of orphans. The main objective of the study is to examine the relationship between care types and development of orphans in China. Furthermore, we want to explore what factors are influencing the effectiveness of orphan care.

*3. Explain why this issue is important and identify the gaps or shortcomings in previous research that this study aims to address.*

We appreciate your thoughtful inquiry. As we mentioned in the paper, in China, orphan care includes both relative and non-relative forms of parenting, as well as family-based and institutional-based care. Different care types have different effects on the development of orphans. However, there are few studies evaluating the effectiveness of different care types. Therefore, this paper tends to explore the impact of the care types on the development of orphans. Besides，the gaps or shortcomings in previous research have primarily centered on two key aspects. First, there is a lack of sufficient academic discussion on the factors contributing to the varying effects of different care types. Second, academic debate continues regarding whether different care types have distinct impacts on children across various groups.

We have supplemented and explained your suggestions regarding the introduction in this revised manuscript. We have modified the manuscript and also marked it in red in the manuscript (lines 76-77).

***Discussion***

*1. In the discussion, avoid writing the table as in Table 4 Impact of placement type on the development of orphans of different genders and education levels*

We thank the reviewer for this constructive suggestion, which helped us enhance the quality of the article. In response to your review comments, we have made structural adjustments to the content related to Table 4. We have incorporated most of the revised content into the conclusion section to present the results of the heterogeneity analysis by gender and age. Additionally, we have expanded the discussion section to further explore the relationship between our findings and previous academic research. The specific adjustments have been marked in red (lines 227-229，234-238，273-282) in the manuscript.

*2. Discuss how your results relate to findings from previous relevant research. Identify any similarities, differences or contradictions between your results and previous research.*

We sincerely appreciate the reviewer's thorough examination of our article. This paper supports the exploration of the relationship between care types and orphan development. We examined the impact of different care types on orphan development, as well as other factors influencing their growth across various care settings and the differences among them. The research findings indicate three parts. First, the results of this study show that the social participation of guardians has a moderating effect on the influence of care types on the development of orphans. Social participation plays an important role in the social isolation and vulnerable group of orphans and can promote the change of family resilience (Lin,2018). Therefore, when grandparents' social participation is increased, its positive impact on the development of orphans will also be strengthened. Second, compared with boys, care types has a greater impact on girls' development. The reason, combined with existing studies, may be explained as follows: on the one hand, girls care more about emotional quality than boys, their emotional needs are more sensitive than boys (Wang Su et al., 2020). Third, for orphans at different stages of education, the influence of care tpyes has obvious heterogeneity. The significant association between grandparents and child development occurs at a young age, and the influence of guardians on orphans diminishes with age (Li et al., 2022). Therefore, children in compulsory education are more vulnerable to grandparents and their social participation than children in non-compulsory education (Lin,2018). The specific adjustments have been marked in red (lines 227-229，234-238，273-282) in the manuscript.

***Conclusions***

*The conclusion has clearly presented the main findings of the research. However, it needs to be revised to improve clarity and consistency to make it easier for readers to understand the main findings and implications of your research.*

We thank this suggestion from the reviewer, which makes the conclusion more clearer. Following the principles of clarity, consistency, and other guidelines you suggested, we have rewritten the entire conclusion section. The new conclusion section is as follows:

By studying the impact of care types on the development of orphans in China, we found that grandparent care can promote the development of orphans. We also found that this impact was moderated by the social participation of guardians. The higher the social participation of guardians, the greater the positive impact of grandparent care on the development of orphans. Moreover, empirical research suggested that girls and younger orphans tend to exhibit better development in a grandparent care family.

Grandparent care is the predominant type of care for orphans’ care in China. Considering the economic situation and labor capacity of grandparents, more support, such as subsidies, should be provided to families where grandparents are the primary caregivers. The government should also ensure the implementation of relevant policy documents on orphan placement and protect the rights of grandparents as primary guardians.

The specific adjustments have been marked in red (lines 333-341) in the manuscript.

Once again, we appreciate the constructive guidance from the reviewer, and we are committed to implementing these valuable insights to enhance the clarity and focus of our paper.
